# Neurological manifestations of the West Nile virus infection in some cases found in the clinic of infectious diseases in Sibiu, Romania

**DOI:** 10.1186/1471-2334-14-S7-P47

**Published:** 2014-10-15

**Authors:** Victoria Bîrluțiu, Rareş-Mircea Bîrluțiu

**Affiliations:** 1 “Lucian Blaga” University Sibiu, Romania; 2 Academic Emergency Hospital Sibiu, Romania

## Background

The neurological manifestations in the West Nile virus infections are present in less than 1% of the diagnosed cases, neurological manifestations which may take many different forms, from encephalitis, meningitis, Guillain-Barré syndrome, optic neuritis to polio-like manifestations. We aimed to evaluate the clinical and paraclinical aspects of the cases presenting neurological infections caused by the West Nile virus diagnosed and treated in our clinic.

## Methods

From 2010 to 2013 a total number of 7 cases with the West Nile neurological infection were confirmed by the presence of specific immunoglobulins M in the serum and the cerebrospinal fluid. The cases were evaluated in terms of demographic data, associated diseases, clinical manifestations, CSF (cerebrospinal fluid) modifications and imagistic studies.

## Results

Four patients were female and 3 were male, aged between 24 and 57. In one case the onset was in Spain. The onset took the form of cerebellum ataxia associated with fever in 4 cases, digestive manifestations in other 4 cases and 1 case presented flu-like syndrome. 3 patients associated morbilliform rash, mostly on the lumbosacral region. The neurological manifestations were dominated by dysmetria, tremor, nystagmus, walking disorders and balance disorders in 4 cases, one of which with opsoclonus myoclonus syndrome (OMS), palsy in 3 cases and tonic-clonic generalized seizure in 1 case. The laboratory exams showed an increased number of white blood cells (WBC) with neutrophilia in 5 out of 7 cases, increased C reactive protein and fibrinogen in 3 patients. The CSF study showed a low number of elements (between 22 and 134 elements/cmm), elevated glycorrhachia in 6 cases, CSF proteins lower than 1 g/dL in 6 cases, nucleated cells – granulocytes 25-56% in 5 cases. The imagistic studies presented specific modifications for acute ischemic stroke in 2 cases. The patient with OMS had an unfavorable evolution despite the treatment.

## Conclusion

The neuro-infections caused by the West Nile virus had severe clinical manifestations, dominated by cerebellum ataxia and palsy. The CSF study presented an increased number of granulocytes that can induce problems regarding the differential diagnoses with bacterial meningitis decapitated by antibiotics or with bacillary meningitis. Hyperglycorrhachia was associated also in patients with normal values of glycemia. OMS associated with the West Nile virus infection is described, in literature, only in a few cases.

